# Rare presentation of subcapsular hepatic steatosis in a woman with uncontrolled diabetes without peritoneal dialysis: a case report

**DOI:** 10.1186/s13256-016-1152-8

**Published:** 2016-12-20

**Authors:** Varun Chowdhary, Jennifer S. Golia Pernicka, Richa Sharma

**Affiliations:** Department of Radiology, Staten Island University Hospital, Northwell Health, 475 Seaview Ave, New York City, NY 10305 USA

**Keywords:** Subcapsular, Hepatic steatosis, CT, MR, In and out-of-phase, Diabetes, Case report

## Abstract

**Background:**

Subcapsular hepatic steatosis is a rare atypical pattern of fatty deposition of the liver reported in patients with diabetic nephropathy receiving peritoneal dialysis with intraperitoneal insulin. To date, there has been only one pediatric and zero adult cases of subcapsular hepatic steatosis with no history of continuous ambulatory peritoneal dialysis. We report the first published case of subcapsular hepatic steatosis in an adult diabetic patient without any history of peritoneal dialysis or evidence of chronic renal disease.

**Case presentation:**

A 46-year-old Caucasian woman with type 2 diabetes mellitus without renal disease presented to our emergency department with vague abdominal symptoms and vomiting. Her blood glucose levels were poorly controlled with a range of 400 to 500 mg/dL. She was diagnosed as having subcapsular hepatic steatosis based on magnetic resonance imaging. Of note, after improved glucose control her subcapsular hepatic steatosis had nearly resolved.

**Conclusions:**

Subcapsular hepatic steatosis has been exclusively described in patients with continuous ambulatory peritoneal dialysis and those on intraperitoneal insulin, except for one pediatric case, which was probably due to incorrect insulin administration. Our case demonstrates that a diagnosis of subcapsular hepatic diagnosis should not be restricted to those getting continuous ambulatory peritoneal dialysis, but rather expanded to all patients with uncontrolled blood glucose levels.

## Background

Subcapsular hepatic steatosis (SHS) is an unusual and rare pattern of fatty deposition of the liver that was first described by Wanless *et al*. in 1989 [1]. With the exception of one pediatric case, all previously published cases of SHS have been associated with continuous ambulatory peritoneal dialysis (CAPD) with intraperitoneal insulin. One theory for the unusual pattern of SHS is that CAPD exposes the subcapsular hepatocytes to a higher concentration of insulin than the remainder of the liver. Subsequently, the insulin blocks the oxidation of free fatty acids in the hepatocytes and leads to preferential esterification of triglycerides which then accumulate within the cell [2].

The most common patterns of hepatic steatosis are diffuse versus focal depositions adjacent to the falciform ligament or gallbladder fossa [3]. However, several cases of atypical distribution have been described in the literature. In particular, patients with diabetic nephropathy on CAPD with intraperitoneal insulin have been shown to have a rare pattern of fat deposition that can be seen on computed tomography (CT) and magnetic resonance imaging (MRI). The pattern is often described as discrete and nodular subcapsular hypoattenuating lesions [2]. Although there has been no clinical adverse effect reported from this unusual fatty infiltration, it is important to understand this entity in order to differentiate it from other similarly low attenuation lesions such as metastatic cancer, primary infiltrative neoplasms, hamartomatous lesions, hematomas, or abscesses [3, 4].

## Case presentation

**Fig. 1:**
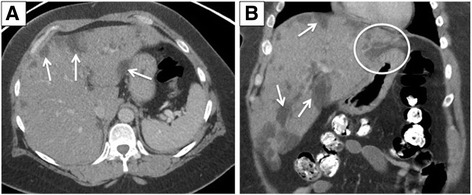
Contrast-enhanced computed tomography of the liver in (**a**) axial and (**b**) coronal views. Multiple hypodense lesions are noted throughout the liver in a subcapsular distribution (*arrows*) as well as perivascular (*circle*)

**Fig. 2:**
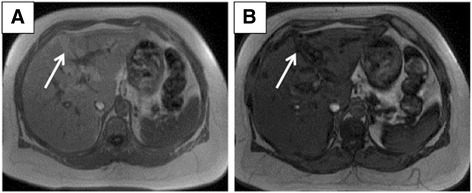
Axial images through the liver confirm subcapsular steatosis as seen on computed tomography in Fig. 1. **a** Axial T1-weighted in-phase gradient echo magnetic resonance imaging (repetition time/echo time = 150/4.4) shows areas of hyperintensity in the subcapsular region (*arrow*). **b** Axial T1-weighted opposed-phase gradient echo magnetic resonance imaging (repetition time/echo time = 150/2.2) shows signal loss in the corresponding subcapsular regions (*arrow*)

**Fig. 3:**
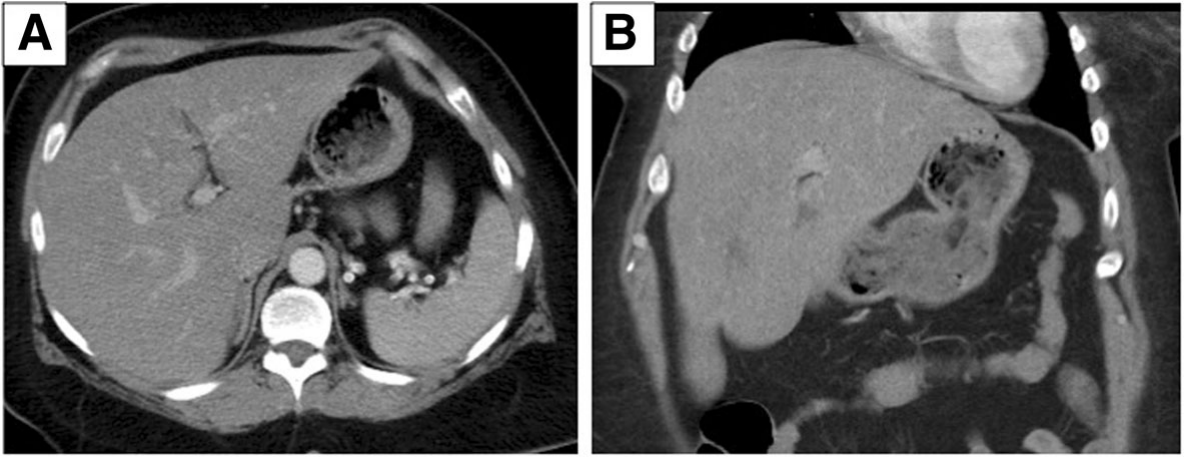
Follow-up scan 3 months after the initial study (Fig. 1) demonstrates resolution of the geographic hypoattenuating areas. The patient underwent a stricter insulin regimen and education to reduce her average blood glucose levels. **a** Axial and **b** coronal views are shown

A 46-year-old Caucasian woman with type 2 diabetes mellitus and bipolar disorder presented to our emergency department with vague abdominal symptoms and vomiting. Her pertinent history includes left below knee amputation and right toes amputation for complications secondary to diabetic neuropathy. At the time of admission, she was undergoing care for an infected diabetic ulcer of her right foot. Of note, she did not have a history of CAPD or a history of renal disease: creatinine 1.23 mg/dL, blood urea nitrogen (BUN) 16 mg/dL. Her blood glucose levels were poorly controlled via subcutaneous insulin injection; she reported a range of 400 to 500 mg/dL at home (due to poor drug compliance). Her blood glucose levels were decreased to a range of 175 to 378 mg/dL after implementation of a stricter insulin regimen upon admission. A non-contrast CT scan showed confluent, bilobar geographic regions of hypoattenuation in a subcapsular distribution throughout her liver (Fig. 1). A MRI liver protocol was performed for further evaluation of these indeterminate findings to assess for possible vascular etiology as areas of infarction could also be possible in this patient. In-phase gradient echo images demonstrated hyperintense foci in her liver in a distribution corresponding to the hypoattenuating regions seen on CT. On the opposed-phase sequence, there was loss in signal within these areas indicating the presence of intracellular fat and water (Fig. 2). In addition, these areas were hypointense to the remaining hepatic parenchyma on the fat suppression MR sequences, confirming presence of fat and thus establishing a diagnosis of SHS. Furthermore, a follow-up CT of her abdomen and pelvis was performed 3 months later, which showed near complete resolution of these findings (Fig. 3). Of note, stricter glucose control had decreased her average blood glucose level to below 200 mg/dL.

## Discussion

Hepatic steatosis is characterized histologically by triglyceride accumulation within the cytoplasm of hepatocytes. The two most common causes are associated with alcohol-related liver disease and insulin resistance/metabolic syndrome. It is very common to diagnose hepatic steatosis on cross-sectional imaging of the abdomen, as the prevalence is approximately 15% in the general population [5]. It is important for the radiologist to understand both the typical and atypical pattern of fat accumulation as some may mimic neoplastic disease, inflammatory changes, or vascular infarcts. Patients are generally asymptomatic.

Although the gold standard of diagnosing hepatic steatosis is liver biopsy, the diagnosis of fatty liver can confidently be made with imaging. Diffuse fat deposition in the liver is the most frequently encountered pattern; however, there are four other main types: focal deposition and focal sparing, multifocal deposition, perivascular deposition, and subcapsular deposition [5]. Focal deposition and focal sparing characteristically occur adjacent to the falciform ligament, in the porta hepatis, and adjacent to the gallbladder fossa. Multifocal deposition is an uncommon pattern where multiple round or oval fat foci are scattered throughout the liver and may mimic true nodules. Perivascular deposition is characterized by halos of fat that surround the hepatic vasculature and create a tram-like or tubular pattern. The last type is a subcapsular deposition pattern, which has been described in the literature with patient on CAPD with insulin added to the dialysate.

Other than one pediatric case in 2012, SHS has been exclusively described in patients with CAPD [6]. Ours is the first adult case of SHS without any history of CAPD or other forms of intraperitoneal insulin therapy. As described by Choh [6], one rationale for the pathogenesis of this pattern of fatty liver without CAPD is that it could be secondary to an incorrect technique of insulin delivery to the anterior abdominal wall, thereby inadvertently creating intraperitoneal spillage. Although possible, this is unlikely in our patient given her body habitus and noncompliance with medications.

One significant differential of SHS is hepatic infarctions. An important factor that would differentiate this entity from SHS is vessels coursing through the hypoattenuated areas, making an infarct less likely. Although this finding was present in our case, our patient underwent an abdominal MRI scan (Fig. 2) which confirmed that the hypoattenuated regions were composed of fat.

## Conclusions

Hepatic steatosis is a common entity in the general population. The incidence increases in patients with hyperlipidemia, hypercholesterolemia, obesity, and diabetes. It is important to understand the various patterns of hepatic steatosis, as well as their respective clinical correlates. Furthermore, it is important to appreciate that the different patterns of hypoattenuation may mimic other conditions. Although SHS predominantly occurs in patients undergoing CAPD with intraperitoneal insulin, our case demonstrates that the pattern of SHS should be considered in the differential even in patients who have not received CAPD.
